# The liquid–amorphous phase transition, slow dynamics and dynamical heterogeneity for bulk iron: a molecular dynamics simulation

**DOI:** 10.1039/d1ra06394d

**Published:** 2021-10-04

**Authors:** Kien Huu Pham, Trang Thi Thuy Giap

**Affiliations:** Department of Physics, Thainguyen University of Education No. 20 Luong Ngoc Quyen Thainguyen Vietnam kienph@tnue.edu.vn tranggt12@gmail.com

## Abstract

Based on molecular dynamics (MD) simulations, we investigate the liquid–amorphous phase transition, slow dynamic and dynamical heterogeneity (DH) for bulk iron in temperatures ranging 300–2300 K. The structure of obtained models is explored through the pair radial distribution function (PRDF) and simplex statistics. It was shown that the splitting of a PRDF second peak appears when the liquid transforms to an amorphous solid. This feature is originated from the transformation of simplexes from strongly-to weakly-distorted tetrahedron type. Further, we reveal that the diffusivity in the liquid is realized through the local density fluctuations (LDF) which are strongly correlated with each other. The diffusion coefficient is found to be a product of the rate of LDF act and mean square displacement of particles per LDF act. The later quantity mainly contributes to the slow dynamics and DH in the liquid. We found that the mobile atom clusters move during relaxation time, but mobile atoms do not tend to leave their cluster. Our work is expected to contribute a pathway to determine the liquid–amorphous phase transition and DH heterogeneity of bulk metal.

## Abstract 2

Based on molecular dynamics (MD) simulations, we investigate the liquid–amorphous phase transition, slow dynamic and dynamical heterogeneity (DH) for bulk iron in temperatures ranging 300–2300 K.

## Graphical abstract

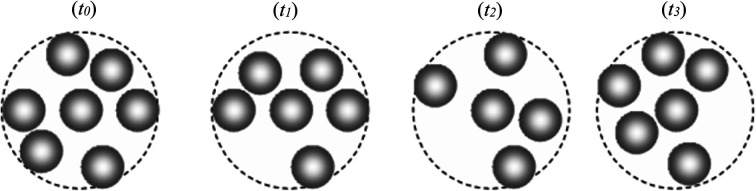

## 1. Introduction

Many liquids might bypass the crystallization and form amorphous solids once the temperature is reduced below their melting point.^1–3^ This transition to a disordered solid known as the glass transition is accompanied by a drastic increase in viscosity and a subtle change in the structure. Understanding the microscopic mechanism governing glass transitions is one of the most important problems in statistical physics.^4–6^ To tackle this problem, several working hypotheses have been proposed. The studies reported in ref. 7–17 focus on the dynamics heterogeneity, the percolation in real space, and properties of energy landscapes. They found the existence of mobile and immobile regions which migrate in space over time. Also, the activation from energy minima is rare at low temperatures, and only infrequent hopping between them causes structural relaxation. Authors in^18–24^ put forward the mechanism by which the small modification of statistic density correlations can produce an extremely large dynamical change. The essential result in this direction is the mode-coupling theory of glass transitions^18^ that predicts the freezing of dynamics from the non-linear feedback effect. The theoretical and experimental investigations on the universal mechanism controlling slow dynamics have been done for a quite amount of time, however as mention in ref. 25 many open questions remained.

Iron (Fe), an element of great interest for industrial application has been under intensive investigation by both computer simulation and experiment.^26–35^ In particular, lots of researchers have been looking closely at the structure and diffusion of liquid Fe.^36–44^ The inter-atomic potentials have been proposed to generate a better result from MD simulation. Recently, the embedded atomic method was widely applied for the metallic system and its alloy.^39,45,46^ However, in some cases, the simulation using the pair potential is most appropriate, because it consumes much less time for computing and reproduces well the experiment results. For example, the MD simulation based on Pak-Doyma potential has been done to explore the structure and dynamics of Fe upon a wide temperature range.^32,40,42,43^ It should be noted that no work relates to the slow dynamics in liquid Fe has been found, and therefore it motivated us to perform a comprehensive simulation on this system using the Pak-Doyma potential.

According to some methods based on the percolation in a real space, a certain number of non-mobile regions can exist at low temperatures where the particle motion is impossible or limited in a small volume. However, when the temperature is adjusted to a lower point, the non-mobile regions are expanded. Upon reaching the melting point they percolated over the whole system that causes slow dynamics. The MD simulation can provide in detail the particle motion with time. Therefore, it allows clarifying the full picture of the non-mobile and mobile regions. This explains extensive MD simulations of slow dynamics within the last two decades. The local density around the particle can be quantified as the ratio of the number of particles in a coordination sphere with radius *R*_0_ to its volume (4π*R*_0_^3^/3). As the coordination number of *i*th particle changes, its local density also varies. It means that the changing of coordination number at some moments can be considered as the event of local density fluctuation (ELDF). In this study, we monitor the ELDF in bulk Fe at different temperatures. Based on the assumption that ELDF occurs rarely in non-mobile regions and happens frequently in mobile regions we will examine how the percolation of non-mobile regions affects the dynamics in the liquid. After that, we identify the reason which causes the slow dynamics and DH in the liquid Fe near the glass transition point. The structure of obtained liquids and amorphous solids has been analyzed through the pair radial distribution function and simplex characteristics.

## 2. Calculation procedure

It is well known that it is very important to select reliable interatomic potentials for MD simulations. Although various interatomic potentials have been proposed for metallic Fe, such as Stillinger-Weber type potentials,^36^ many-body potentials,^37,38^ embedded atom potential (EAM).^39,45^ However, the simple pair potential proposed by Pak-Doyama,^40^ which is no longer popular, this potential well describes the melting temperature, structural and thermodynamic properties of both liquid and amorphous Fe. Indeed, various MD simulations have been carried out using the Pak-Doyama potential to confirm these points.^32,40–43^ In addition, the melting temperature range obtained by heating the bcc crystal is 1920–1965 K.^43^ Due to the overheating achieved in the simulation, the melting point is not much higher than the experimental value of 1811 K.^44^ Generally, the MD model constructed by the Pak-Doyama potential has reasonable consistency with the experiments of the melting, structure, thermodynamic properties and the glass transition temperature of Fe. Thus, in the present work, we use the Pak-Doyama potential which is given as follows

1 *V*(*r*_*ij*_) = −0.188917(1.82709 − *r*_*ij*_)^4^ + 1.70192(*r*_*ij*_ − 2.50849)^2^ − 0.198294;  *r*_*ij*_ ≤ 3.44 Å

here *r*_*ij*_ is the inter-atomic distance in Å and *V*(*r*_*ij*_) in eV. Within a cube containing 10 000 particles, a simulation is carried out under periodic boundary conditions. By using the Verlet algorithm, we numerically tackled the equations of motion. To erase the effect of the initial state on the system, the model was equilibrated at a constant density of 7.1 g cm^−3^ by relaxation for 10^6^ MD steps at a temperature of 5000 K and the ambient pressure in the microcanonical *NVT* ensemble. From this melting point, eight models are prepared at the temperatures from 2300 to 300 K by cooling down until we reached the wanted density and temperature, *i.e.*, the constructed models at 2300, 2050, 1820, 1550, 1200, 850, 650, and 300 K and the ambient pressure. The characteristics of obtained models are listed in Table 1. At selected density/size and temperatures, a long relaxation for each temperature point has been done in an isothermal–isobaric *NPT* ensemble (about 2.5 × 10^7^ MD steps which is equal to 0.4 fs) to equilibrate the model. As a result, the constructed models have different temperatures and near ambient pressure. After that, to collect the dynamical and structural data we also performed additional runs for each equilibrated model within 5 × 10^6^ MD steps in the microcanonical *NVE* ensemble. We use the cutoff distance *R*_Fe–Fe_ = 3.35 Å chosen as a minimum after the first peak of radial distribution function (PRDF) for computing the coordination number.

**Table 1:** The characteristics of the constructed models: *T* – the average temperature of the model; *L* – the length of the simulated cube; *ε* – the average energy of each atom

*T* (K)	2300	2050	1820	1550	1200	850	650	300
*L* (Å)	53.3582	52.3114	51.6136	50.9869	50.3394	49.9674	49.8875	49.7694
*ε* (eV)	−0.8829	−0.9566	−1.0307	−1.1143	−1.2246	−1.3171	−1.3404	−1.3834

In the simplex analysis, necessary information about local structure will be deduced. Consider four particles in the constructed model which form a tetrahedron. Unless the circumsphere this tetrahedron includes any particle inside, we call the set of four particles considered, as a simplex. Further, we denote *R*_S_ and *a*_Si_ (*i* = 1, 2, 3, 4, 5, 6) to the radius and edge-length of the tetrahedron, respectively. If the radius *R*_S_ is large enough, the simplex has a large space inside and represents a point defect in the disordered structure. Properties of all simplexes detected in the constructed model are determined by two functions: the distribution of radius *R*_S_ and edge-length *a*_Si_.

**Fig. 1:**
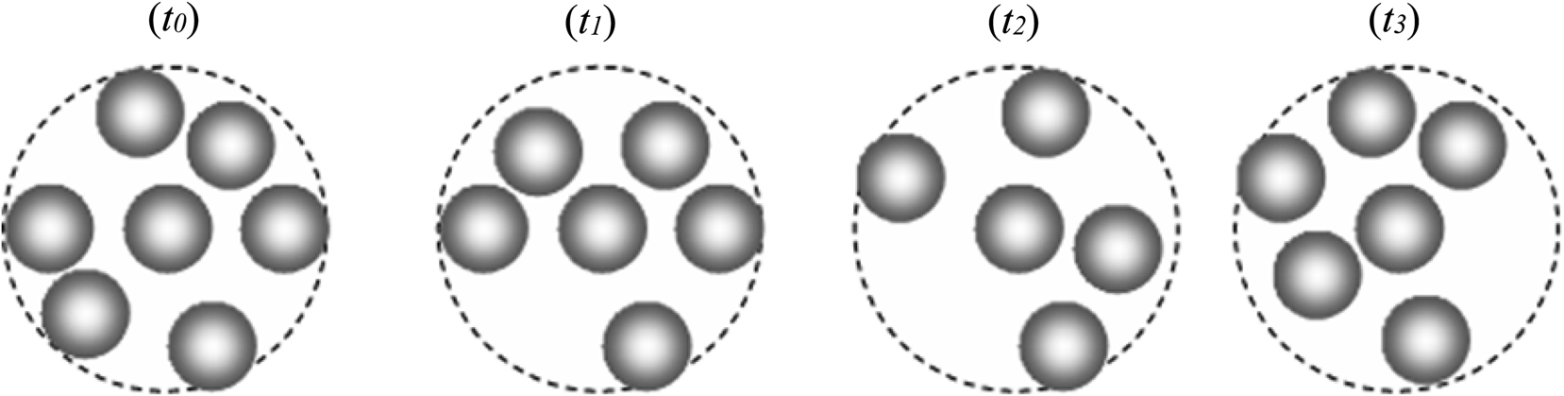
The schematic illustration of the evolution of local density for selected particle; the dash circles represent the coordination sphere of selected particle. During a run, the local density of the selected particle changes four times at moments *t*_0_, *t*_1_, *t*_2_, and *t*_3_ that the number of ELDF is equal to 4.

As we have known, a liquid on cooling can be crystallized or experienced dynamic slowing down leading to glass formation. It currently believes that the slow dynamics of a supercooled liquid is related to the diverging correlation length of cooperative motions, that is, dynamic heterogeneity (DH).^12,47,48^ On the other hand, the dynamics of a system can be measured by the event of local density fluctuation (ELDF). Therefore, to clarify a mechanism for the slow dynamics “dynamic slowing-down”, we analyze the ELDF through the coordination cell which is defined as a set of the central atom and its neighbors. Note that the changing of coordination number of atoms at some moments can be considered as the ELDF. The cutoff distance used to calculate the neighboring atoms is 3.35 Å. This distance is chosen as a minimum after the first peak of PRDF. The ELDF is caused by reactions Fe_*n*_ ↔ Fe_*n*′_, here *n*, *n*′ is the coordination number of Fe atom. Most reactions satisfy the condition |*n* − *n*′| = 1. Other types may have occurred, for instance, Fe_6_ → Fe_8_ → Fe_6_, *i.e.* two Fe atoms enter or leave the coordination cell at the same time. However, this type is extremely rare. During the simulation, we registered coordination cells and determine the number of reactions that happened. The schematic illustration of ELDF for a selected particle is presented in Fig. 1.

## 3. Results and discussion

### 3.1. The liquid-amorphous structural transition

**Fig. 2:**
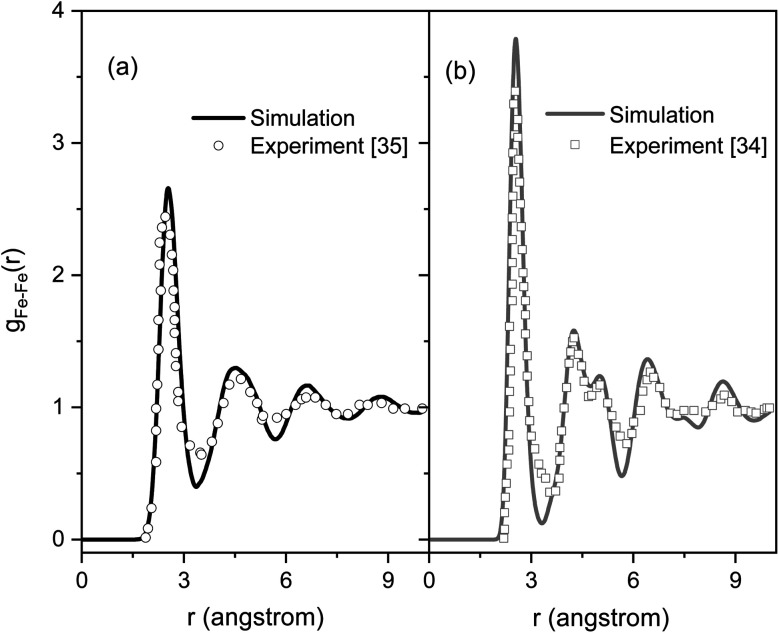
The pair radial distribution functions for liquid and amorphous Fe models at 300 and 1820 K and experimental data:^34,35^ (a) liquid Fe, (b) amorphous Fe.

**Fig. 3:**
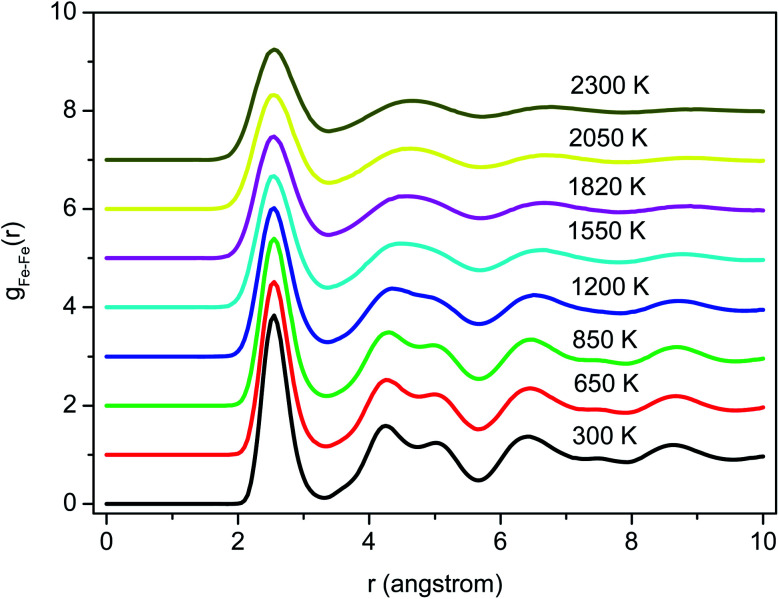
The radial distribution functions for Fe models at different temperatures.

Fig. 2 shows the PRDFs obtained at different temperatures and the comparison with experimental data for liquid and amorphous models.^34,35^ There is a good agreement with the experiment indicating that the Pak-Doyma potential can reproduce well both liquid and amorphous states of Fe. As shown in Fig. 3 the height of PRDF first peak increases with decreasing the temperature, so it follows that the nearest neighboring coordination is raised during solidification of the material. Further, the positions of PRDF first peak and first minimum are almost unchanged with temperature. They are located at 2.55 and 3.35 Å, respectively. It should be noted that below 1200 K the second peak of PRDF is split which shows the transition from the liquid to the amorphous state. The splitting of PRDF second peak is also observed by other researchers for different materials. This result was thought to be related to the existence of local icosahedral order in material^43^ and considered as a common feature of amorphous matter.^34,40,45,46,49^

**Fig. 4:**
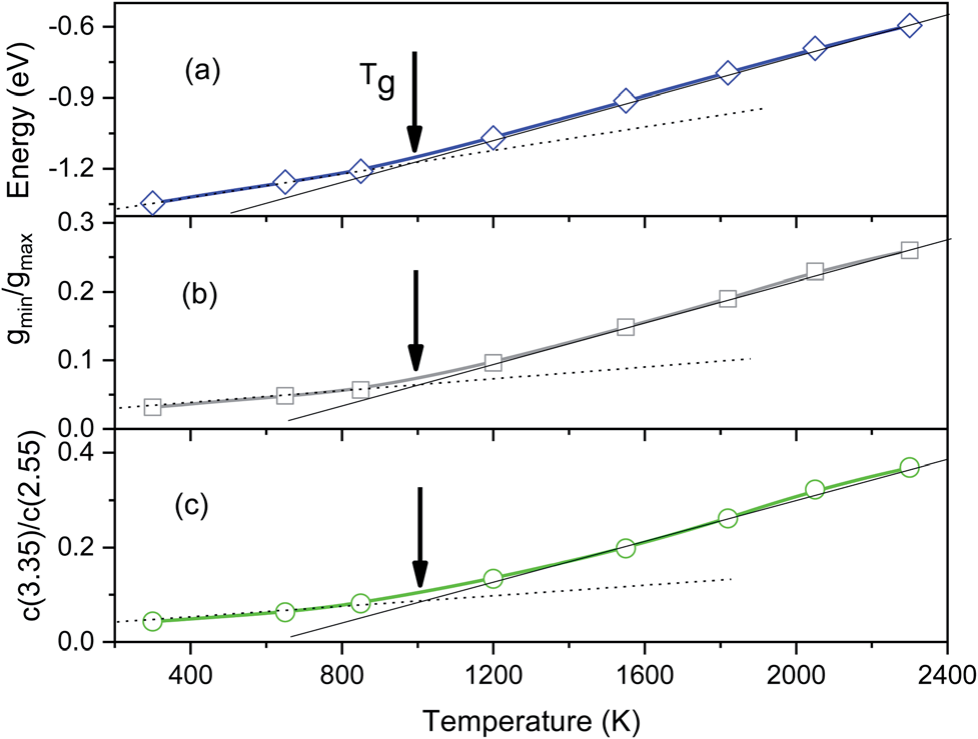
The temperature dependence of the energy (a), the Wendt–Abraham ratio *g*_min_/*g*_max_ (b), and the ratio *C*(3.35)/*C*(2.55) (c).

To determine the glass transition temperature (*T*_g_) in MD simulations we use the Wendt–Abraham ratio defined as *g*_min_/*g*_max_. Here *g*_min_ is the first minimum value and *g*_max_ is the first maximum value of PRDF. The ratio *g*_min_/*g*_max_ at different temperatures is calculated and the obtained data plotting *versus* temperature is shown in Fig. 4. From this figure, the temperature *T*_g_ is obtained near 1080 K which is close to the temperature where the splitting of PRDF second peak starts. Using the mean energy per particle which is given in Fig. 3, we also obtain the glass transition temperature to be 1060 K that less than one determined by the ratio *g*_min_/*g*_max_.

**Fig. 5:**
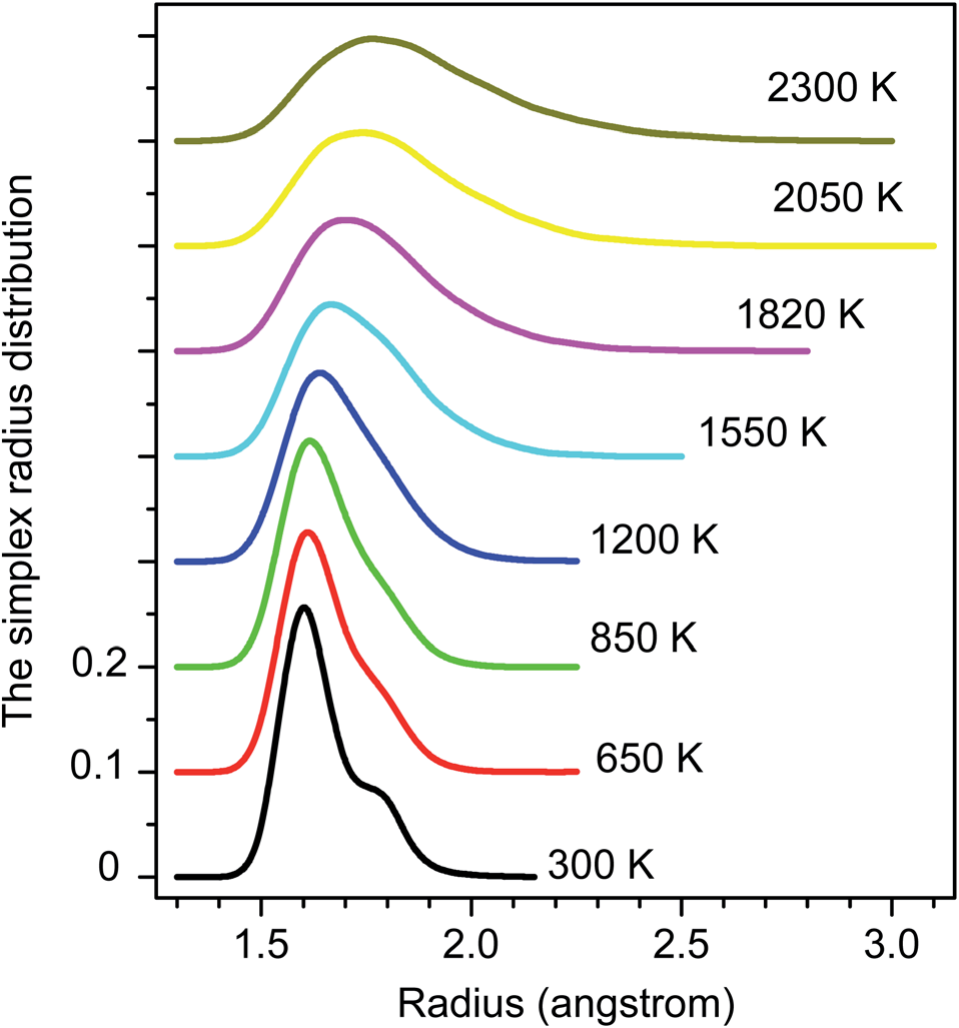
The radius distribution of simplexes.

**Fig. 6:**
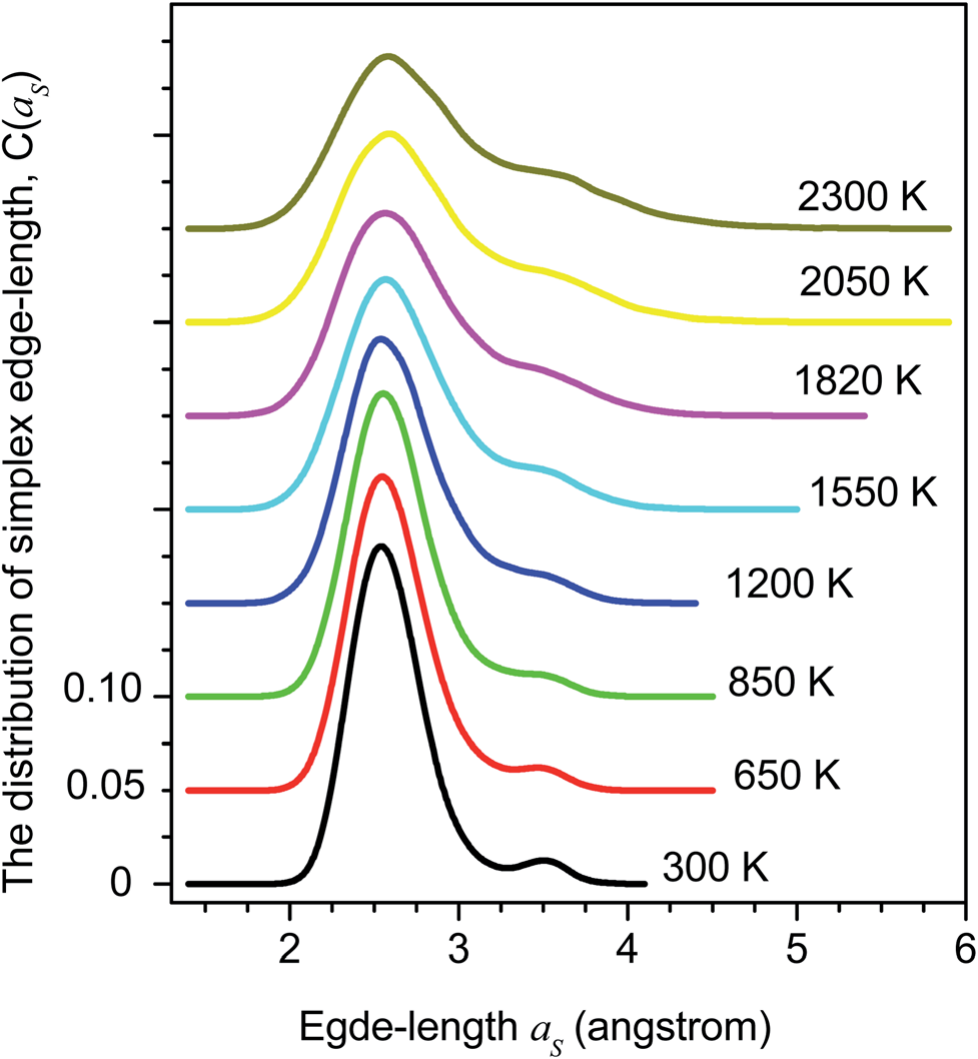
The distribution of simplex edge-length.

The distribution of simplex radius given in Fig. 5 shows the monotonous increase in the height of the main peak with increasing the temperature. In addition, the location of the peak is slightly shifted to the left. Hence the higher the temperature, the larger the simplexes are. It is interesting to note that below 1200 K there is appearing of a plateau. Therefore, the change in simplexes under temperature relates to the structural transformation from liquid to amorphous states, *i.e.* to the splitting of PRDF second peak. This fact can be also seen from the distribution of edge-length shown in Fig. 6. Here we again observe the appearance of sub-peak upon temperature below 1200 K. Note that a right tetrahedron with an edge length of 2.6 Å has a circumsphere with a radius of 1.59 Å. These values are close to the location of the peaks (2.56 and 1.6 Å) shown in Fig. 5 and 6, so we can conclude that the simplexes in amorphous solid are close to the right tetrahedron type, meanwhile, the simplexes of liquid are strongly distorted tetrahedron type. It means that the solidification of Fe accompanies the simplex transformation from right to distorted tetrahedron type. In Fig. 4 we also plot the ratio *C*(3.35)/*C*(2.55) which are taken from Fig. 6 corresponding to the edge-length of 3.35 and 2.55 Å, respectively. One can see that the ratio *C*(3.35)/*C*(2.55) varies like the ratio *g*_min_/*g*_max_ which gives a glass transition point near 1060 K. Note that the locations of the first peak and minimum of PRDF have values of 3.35 and 2.55 Å, respectively. This fact again indicates that the change in simplexes directly relates to the structural transformation from liquid to an amorphous state. As mention above, the transition to an amorphous state accompanies the splitting of PRDF second peak, therefore, the transformation of simplexes from the right to distorted tetrahedron type causes the observed splitting of the second peak.

### 3.2. Slow dynamic and dynamical heterogeneity

**Fig. 7:**
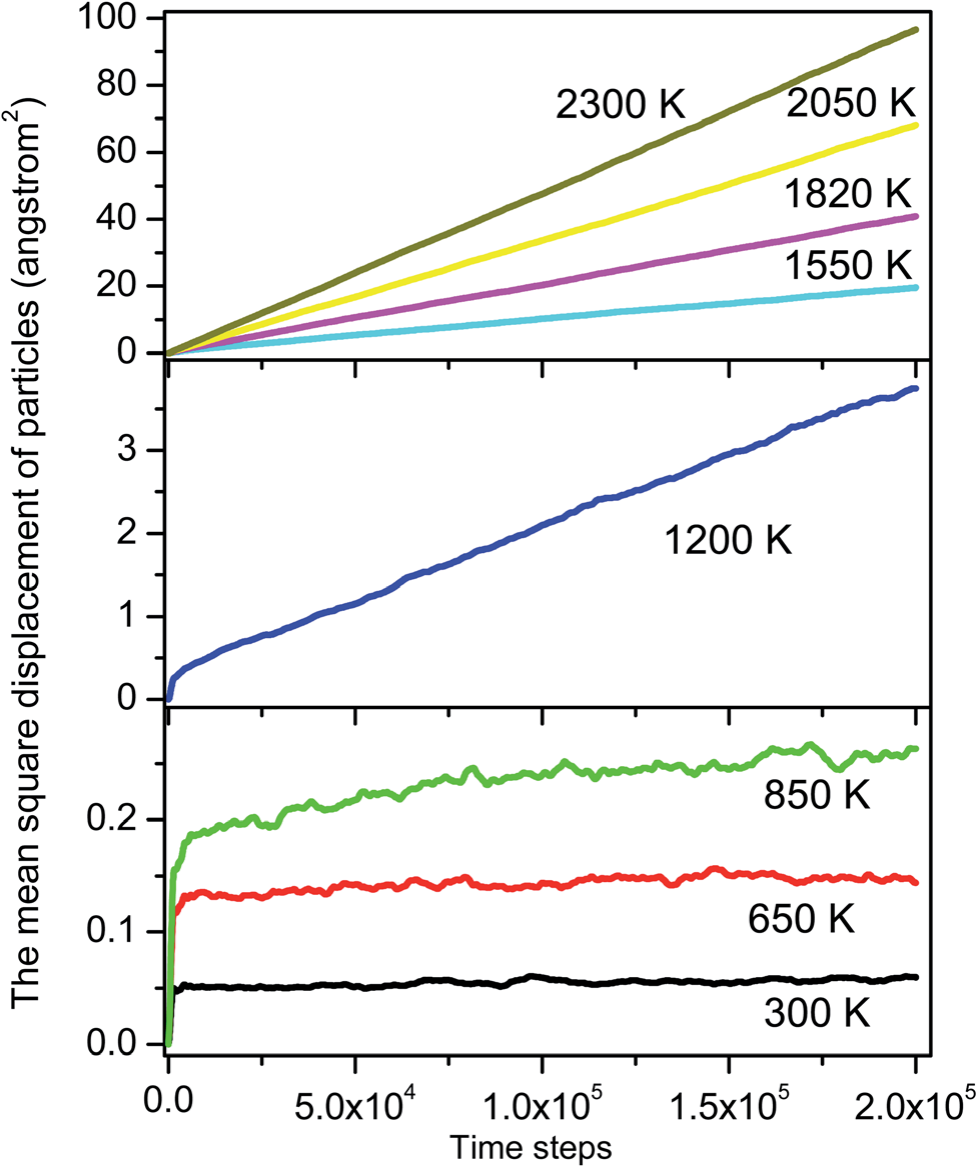
The mean square displacement of all particles.

In MD simulation the diffusion constant has been determined *via* an Einstein relation2
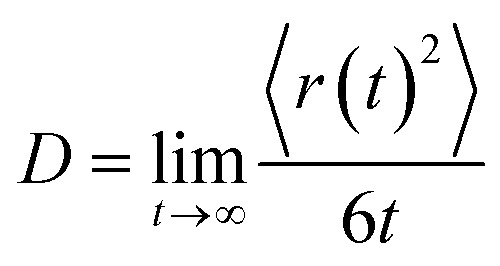
here 〈*r*(*t*)^2^〉 is the mean square displacement of all particles over time *t*. The curves 〈*r*(*t*)^2^〉 *vs.* MD steps shown in Fig. 7 presents well-straight lines for high temperatures. Their slopes are used to calculate the diffusion constant. As the temperature is below 850 K, the graph becomes a horizontal line which shows that the diffusion constant drops to zero due to the material transforms from liquid to an amorphous solid.

**Fig. 8:**
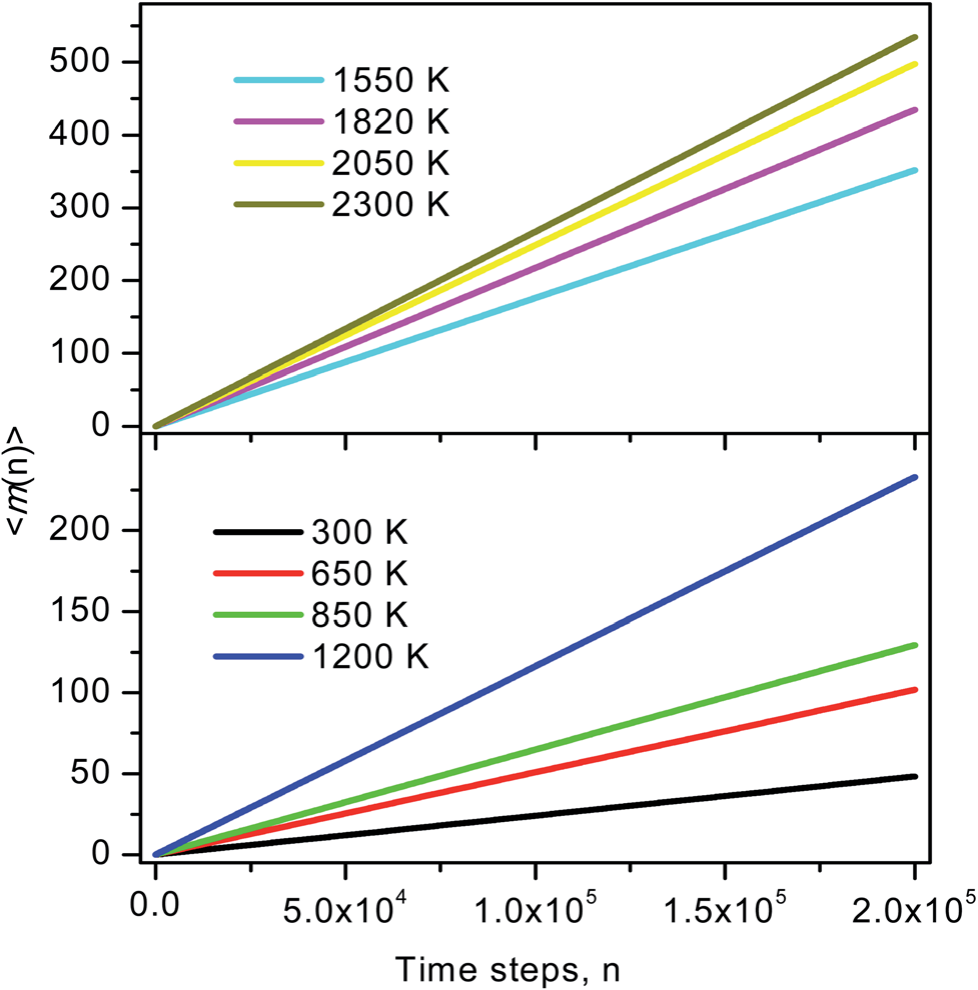
The dependence of 〈*m*(*n*)〉 *versus* MD steps *n*.

**Fig. 9:**
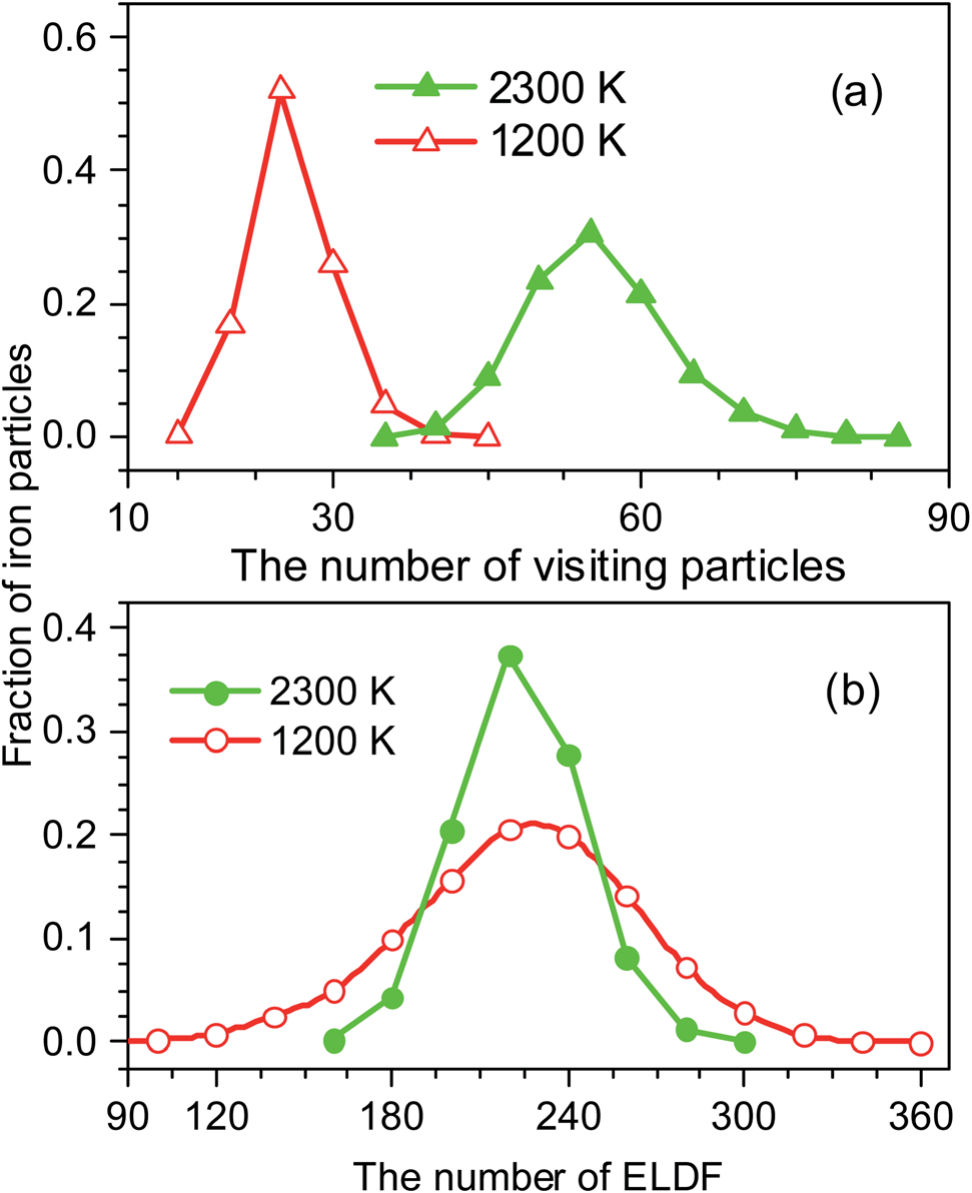
The temperature dependence of the quantities *δ*.

During the simulation run for each particle, we determine the number of the event when a fluctuation of local density (ELDF) occurs. Let *m*_*i*_(*n*) is several ELDF happening with ith particle during *n* MD steps. The quantity of interest is the mean number of ELDF per particle which is given as3
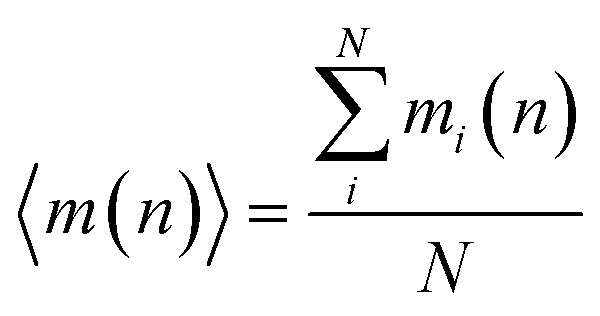
here *N* is the number of particles in the model. The data of 〈*m*(*n*)〉 presented in Fig. 8 shows well-straight lines which slope presents the rate of ELDF, *ξ*. The parameter *ξ* is determined in the unit of the number of ELDF/MD steps. The relation (2) is transformed to4
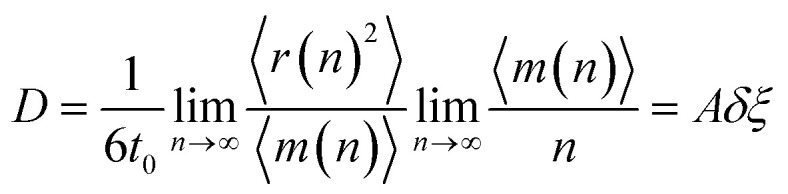
where *A* is the constant equal to 1/6*t*_0_, *t*_0_ is the time consumed for one MD step; *δ* is the mean square displacement of all particles as one ELDF occurs, which is in the unit of Å^2^/one ELDF. The obtained *δ* are presented in Fig. 9. As seen, the parameter *δ* rapidly reduces to zero with decreasing the temperature. This result presented that *δ* mainly contributes to the slow dynamics near the glass transition point. It means that significant variation of *δ* value indicates ELDF causes different collective movements of particles at different temperatures.

**Fig. 10:**
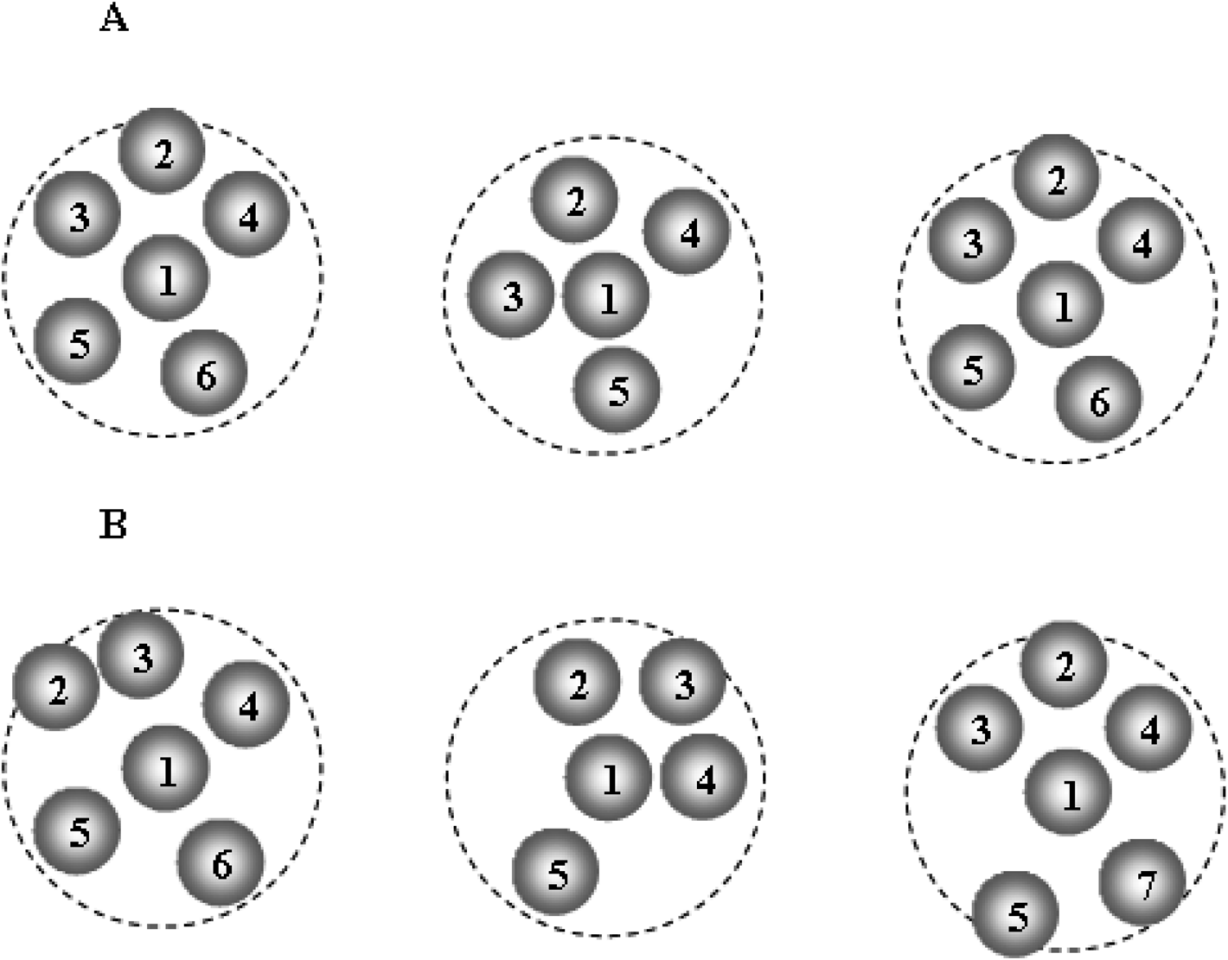
The number of visiting particles (a), and the distribution of ELDF (b).

To identify these movements we perform two runs for models at the temperature of 1200 and 2300 K. For each run the number of MD steps *n* is adopted so that the total number of ELDF, ∑*m*_*i*_(*n*) is close to a given value. The value of ∑*m*_*i*_(*n*) is 233174 and 2331573 for models at 1200 and 2225 K, respectively. Fig. 10 shows the distribution of *m*_*i*_(*n*) through particles for considered models. There is a pronounced peak which location is almost unchanged with temperature. The curves have a Gauss form. Its height for a low-temperature run is lower than for a high-temperature one. Moreover, the graph for a low-temperature model is much wider. This observation can be explained by the assumption that ELDF is realized by the activation from different energy barriers. The energetic barrier set is not changed significantly under temperature. Hence, at a low temperature ELDF happens more frequently for the particles where the activation energy is small, whereas it is rare where the activation energy is large. As a result, the distribution of *m*_*i*_(*n*) is spread wider with decreasing the temperature. It should be noted that the redistribution of ELDFs in a low-temperature model does not lead to the formation of regions where ELDFs happen rarely and those regions percolated over whole systems. We observe the homogeneous distribution of ELDF in the space for both low- and high-temperature models. Therefore, according to our simulation, the slow dynamics mechanism near the glass transition point can be described as follows: (i) at higher temperatures, some small slow regions containing the “slow atoms” form in different places of liquid and dissolve for short times; (ii) as temperature decreases, small slow regions form and gather nearby which creates the stable slow dynamic region in the system. After that, the stable slow dynamic regions grow with decreasing temperature, and near the glass transition point, the slow dynamic regions are expanded the whole system.

**Fig. 11:**
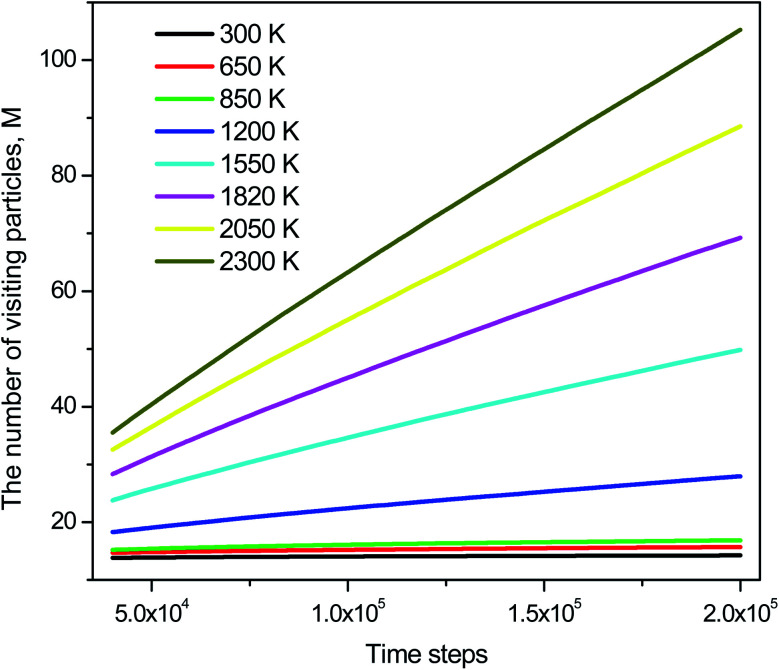
The schematic illustration of two types of ELDF; (A)/effective ELDF; (B)/non-effective ELDF.

Fig. 11 presents the schematic illustration of ELDF occurring in the constructed models. One can see two types of ELDF. For the first type after ELDF happened, particle 6 leaves the coordination sphere, and then it comes back as the next ELDF occurs. As a consequence of two ELDFs the list of particles visiting the coordination sphere is unchanged. In the case of the second type, the list of particles visiting the coordination sphere contains also particle 7, because particle 7 enters the coordination sphere replacing particle 6. The first type is denoted to effective ELDF, and the second to non-effective one. The effective ELDF causes a larger square displacement of particles than non-effective ELDF because in the later case more particles move forth back in the system. To estimate the fraction of effective ELDF during simulation for each ith particle we determine a list of particles visiting the coordination sphere of ith particle. Let the number of particles of this list during *n* MD steps be *s*_*i*_(*n*). The quantity of interest is the mean number of visiting particles is given as5
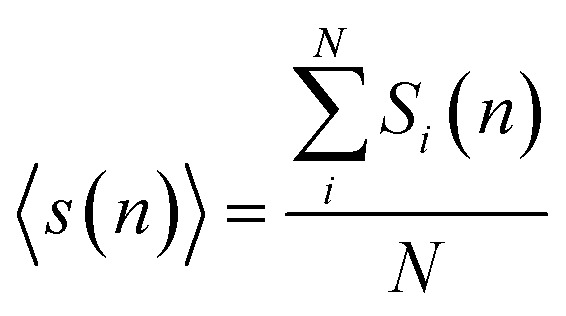


**Fig. 12:**
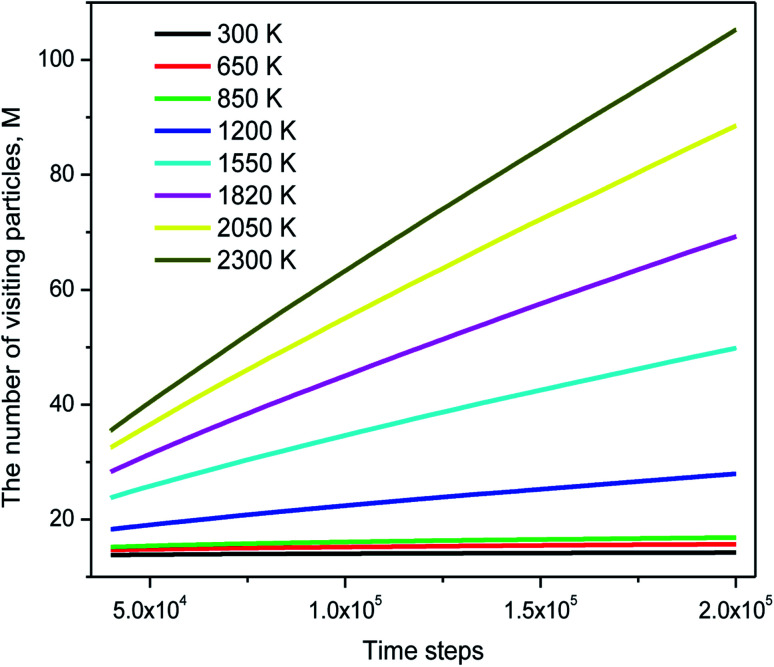
The dependence of the number of visiting particles *vs.* MD step.

The distribution of *s*_*i*_(*n*) is shown in Fig. 10. Unlike the case of *m*_*i*_(*n*) the curve for a high-temperature model is spread wider and the location of the peak is significantly shifted to the left with increasing the temperature. In particular, the peak location is 26 and 54 for low- and high-temperature models, respectively. This result indicates that the fraction of non-effective ELDF significantly increases as the temperature reduces. To this end, we evaluate the dependence of 〈*s*(*n*)〉 *vs. n* for constructed models. The obtained result is presented in Fig. 12. Here one can see that the graph is well-straight lines and their slope increases with temperature. For the low-temperature model, the curve is horizontal indicating all ELDFs are non-effective. Combined these results we can conclude that the diffusion is realized *via* activated ELDFs which do not happen randomly in the space. At low temperatures, they are strongly correlated leading to a large number of non-effective ELDF. In a high-temperature regime, the effective ELDFs are dominant. This means that the DH observed in our simulation is not originated from the percolation of non-mobile regions as shown for the case of network-forming liquids. In converse, the reason concerns the correlation effect that the ELDF are strongly correlated at low temperature.

**Fig. 13:**
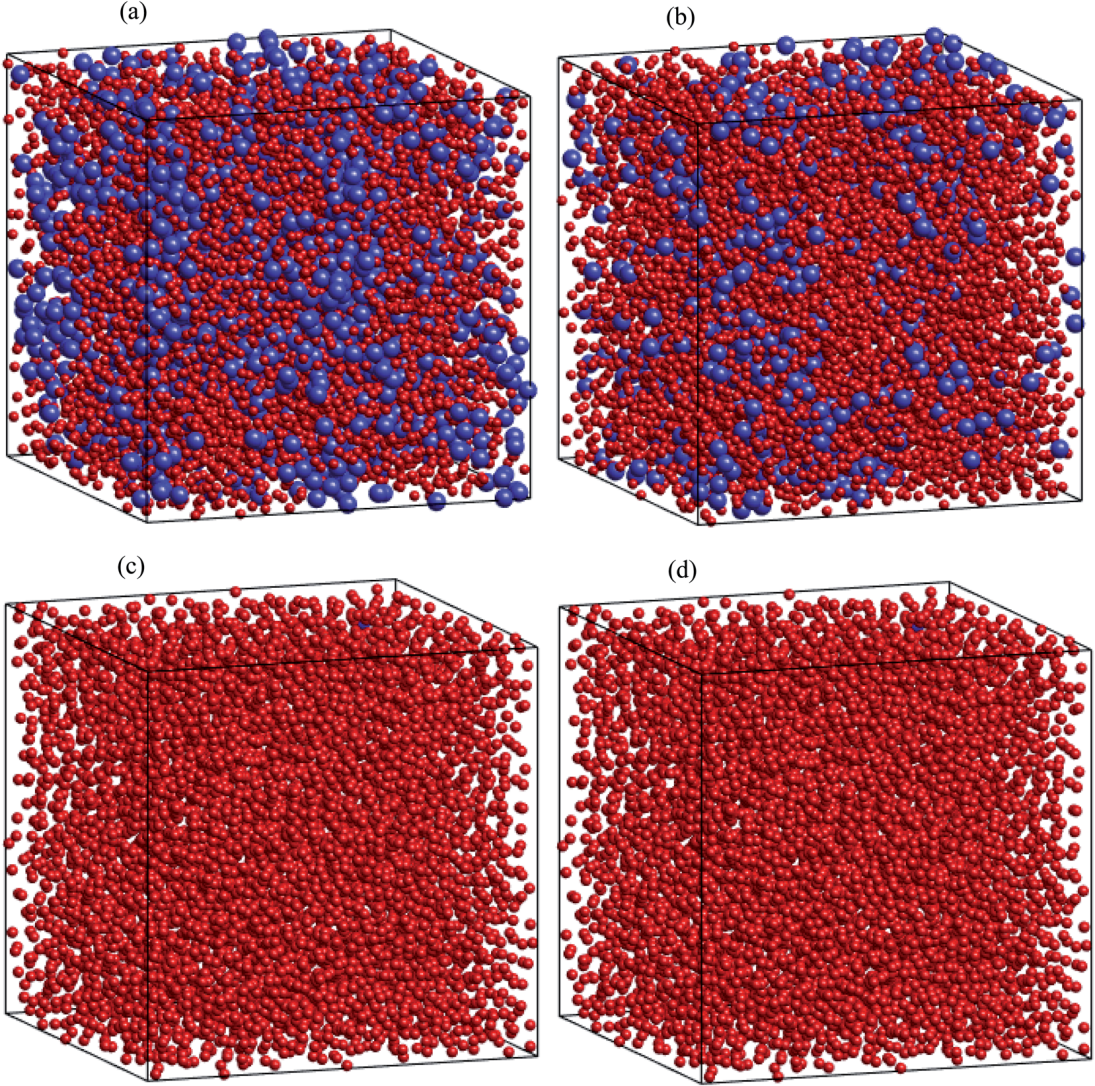
Snapshots of distribution of mobile and immobile atoms at different temperatures: (a) 2300 K, (b) 2050 K, (c) 1200 K, and (d) 300 K in 3D space: here, mobile and immobile atoms are in blue and red balls, respectively.

**Fig. 14:**
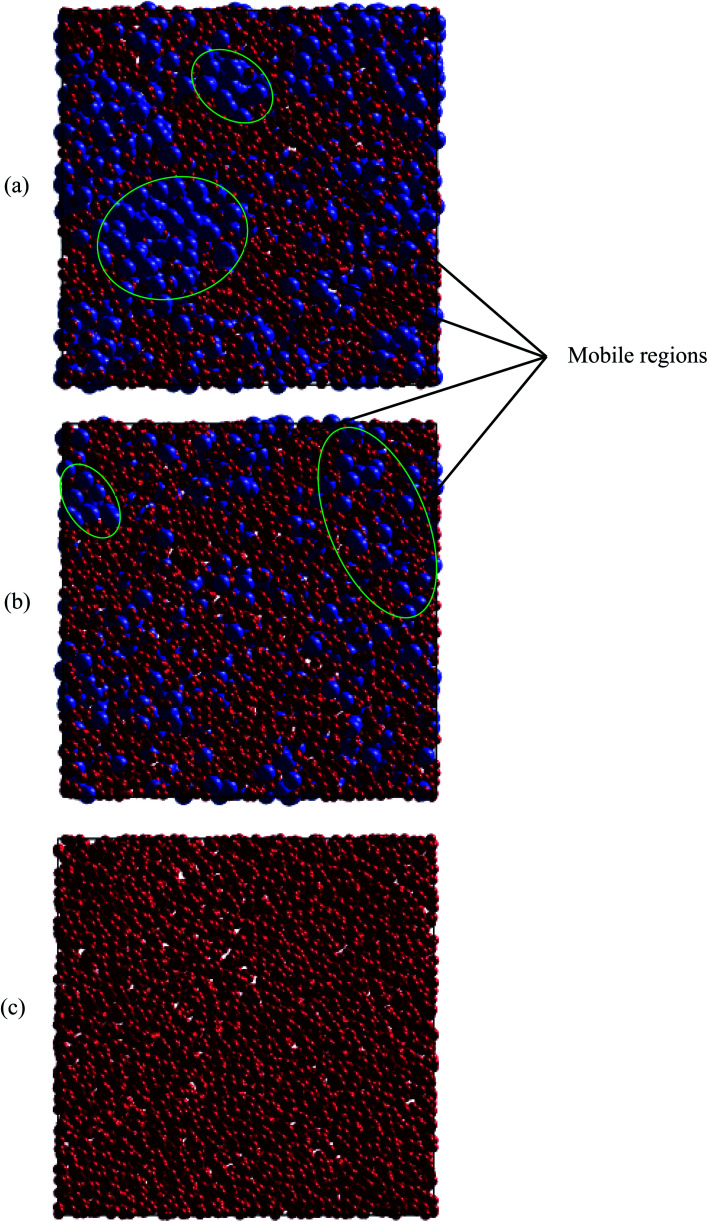
Snapshots of cluster formation of mobile and immobile atoms in a cross-sectional view of the 3D result, at different temperatures: (a) 2300 K, (b) 2050 K, and (c) 1200 K. Here, mobile and immobile atoms are in blue and red balls, respectively.

**Fig. 15:**
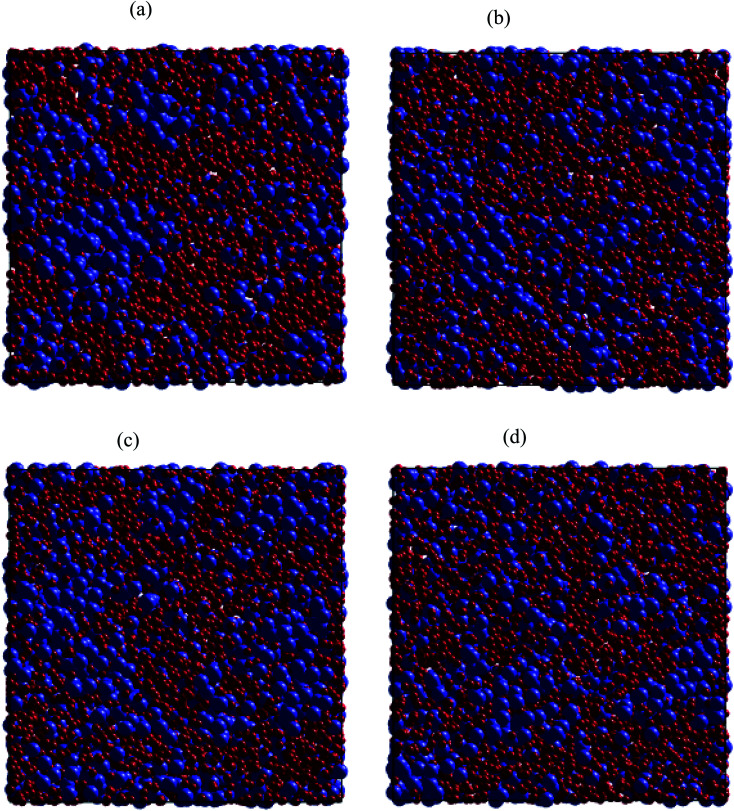
Snapshots of mobile and immobile regions in a cross-sectional view of the 3D result, under different relaxation degrees for Fe liquid at 2300 K: (a) 2 × 10^6^ steps, (b) 6 × 10^6^ steps, (c) 10^7^ steps, and (d) 6 × 10^7^ steps. Here, mobile and immobile atoms are in blue and red balls, respectively.

Regarding dynamical heterogeneity (DH) in metal materials, most studies on the DH tried to monitor a certain percentage of the mobile or immobile atoms and analyzed their spatial distributions.^30,32,42,50,51^ In the present work, we also adopt the same approach. We characterize the mobility of each atom by calculating their nearest neighbors which are located from the center of the sphere at a distance of 3.44 Å (cutoff radius). These nearest neighbors might form an atoms-cage-like. It is difficult for an atom to be moved its cage as considered atom belongs to the atoms-cage having a lot of nearest neighbors because atoms-cage is rigid to be broken for the tagged atom. Otherwise, the atom can be moved out and hops into a new atoms-cage. In our simulation, we considered two typical atoms confined in atoms-cages: the first, atoms are confined in cage including from 10 to 16 nearest neighbors, they are called immobile atoms. Another one has confined in a cage in which the number of nearest neighbors is smaller than 10, they are called mobile atoms. A more comprehensive picture of mobile and immobile atoms requires knowledge not only of the number but also of their distribution. Here, we use visual examination to consider the distribution of mobile and immobile atoms. Fig. 13 and 14 shows a snapshot of the positions of mobile atoms in bulk Fe annealed at a temperature of 300, 1200, 2050, and 2300 K in 3D and a cross-sectional view of the 3D result. It can be seen that the number of mobiles and immobile atoms strongly depends on temperature. Below a temperature of 1200 K, the structure of models includes main immobile atoms. Meanwhile, the number of mobile atoms powerfully tends to decreases to zero under the temperature decreasing. It means that in the temperature range 1200–300 K the atom transport does not follow the Arrhenius law. In addition, in the high temperature, the distribution of mobile and immobile atoms is non-uniform. As shown in Fig. 13, the mobile atoms tend to form a cluster. Consequently, the number of mobile atoms is grouped into the mobile region in liquid. Meanwhile, immobile atoms formed the immobile regions. This means that liquid Fe comprises two-phase: mobile and immobile phases or low and high densities phases as knew in previous works.^29,32,43^ The existence of separate phases may be evidence of DH in liquid Fe. Note that the separate mobile and immobile phases are not observed in amorphous Fe (at below temperature). These results also agree with the above analysis of the diffusion mechanism of Fe atoms. It is interesting that for liquid Fe at a temperature of 2300 K, the mobile atomic clusters move during relaxation time and mobile atoms do not tend to leave their cluster as can be seen in Fig. 15. Note that the 2D result is a cross-sectional view of the 3D result. We can conclude that the dynamics of liquid Fe is DH during relaxation time. This also demonstrated why in liquid Fe the atom transport does follow the Arrhenius law as a reporter in ref. 16, 31 and 43.

The above analysis results indicated that there is a connection between local structure, liquid–amorphous phase transition, and HD for the bulk Fe. The analysis of the distribution of typical atoms also shows that mobile and immobile atoms tend to segregate into separate regions, *i.e.* low and high densities regions. Clearly, in the regions where local packing densities (immobile region) are higher, the motion of atoms is more difficult. A cooperative rearrangement of mobile atoms and their nearest neighbors might be related to form new mobile regions in liquid. Therefore, our studies are also consistent with obtained results in ref. 23–25, 30, 50 and 51.

## 4. Conclusion

In this study, a series of models of the liquid and amorphous Fe in a wide temperature range and ambient pressure has been successfully constructed by MD simulation. The structural analysis of obtained models shows that the position of the main peak of PRDF is almost unchanged with temperature, but the second peak of PRDF appears to split when the liquid transforms into an amorphous solid. The simplexes in the amorphous Fe are like ideal tetrahedrons, in contrast, they are strongly distorted tetrahedrons in liquid Fe. We determined the glass transition point in three different ways: the variation in the total energy, the ratio *g*_min_/*g*_max_, and *C*(3.35)/*C*(2.55) with temperature. The calculation shows a reasonable agreement between results obtained in different ways. In addition, we found that the splitting of the second peak of the PRDF is originated from the transformation of simplexes from distorted to ideal tetrahedron type, corresponding the material undergoes from liquid to amorphous state. We showed that the diffusivity in liquid Fe is realized by the activated ELDF which is strongly correlated at low temperatures. The diffusion constant is found to be a product of the rate of ELDF *ξ* and mean square displacement per ELDF *δ*. As temperature decreases, quantity *δ* rapidly decreases to zero and mainly contributes to the slow dynamics and DH near the glass transition point. This is a new model for slow dynamics and DH which is originated not from the percolation of immobile regions, but the correlation effect between ELDFs happening in liquid. A distinctive result of this research is that, for liquid Fe, the mobile atom clusters move during relaxation time and mobile atoms do not tend to leave their cluster. This also demonstrated the dynamics of liquid Fe in DH during relaxation time. This work suggests a technique to determine the liquid–amorphous phase transition temperature and dynamics heterogeneity of bulk metal.

## Conflicts of interest

There are no conflicts to declare.

## Supplementary Material
